# A mouse model of hypoplastic left heart syndrome demonstrating left heart hypoplasia and retrograde aortic arch flow

**DOI:** 10.1242/dmm.049077

**Published:** 2021-11-10

**Authors:** Anum Rahman, Taylor DeYoung, Lindsay S. Cahill, Yohan Yee, Sarah K. Debebe, Owen Botelho, Mike Seed, Rajiv R. Chaturvedi, John G. Sled

**Affiliations:** 1 Mouse Imaging Centre, The Hospital for Sick Children, Toronto, ON M5T 3H7, Canada; 2 Translational Medicine, The Hospital for Sick Children, Toronto, ON M5G 1X8, Canada; 3 Department of Medical Biophysics, University of Toronto, Toronto, ON M5G 1L7, Canada; 4 Department of Chemistry, Memorial University of Newfoundland, St John's, NL A1B 3X7, Canada; 5 Division of Pediatric Cardiology, Department of Pediatrics, The Hospital for Sick Children, Toronto, ON M5G 1X8, Canada; 6 Department of Obstetrics and Gynecology, University of Toronto, Toronto, ON M5G 1E2, Canada

**Keywords:** Mouse model, Hypoplastic left heart syndrome, Congenital heart disease, Ultrasound, Fetus, Magnetic resonance imaging

## Abstract

In hypoplastic left heart syndrome (HLHS), the mechanisms leading to left heart hypoplasia and their associated fetal abnormalities are largely unknown. Current animal models have limited utility in resolving these questions as they either do not fully reproduce the cardiac phenotype, do not survive to term and/or have very low disease penetrance. Here, we report the development of a surgically induced mouse model of HLHS that overcomes these limitations. Briefly, we microinjected the fetal left atrium of embryonic day (E)14.5 mice with an embolizing agent under high-frequency ultrasound guidance, which partially blocks blood flow into the left heart and induces hypoplasia. At term (E18.5), all positively embolized mice exhibit retrograde aortic arch flow, non-apex-forming left ventricles and hypoplastic ascending aortas. We thus report the development of the first mouse model of isolated HLHS with a fully penetrant cardiac phenotype and survival to term. Our method allows for the interrogation of previously intractable questions, such as determining the mechanisms of cardiac hypoplasia and fetal abnormalities observed in HLHS, as well as testing of mechanism-based therapies, which are urgently lacking.

## Abstract (precis)

**Summary:** We report the first mouse model of isolated hypoplastic left heart syndrome (HLHS), allowing for the investigation of abnormal cardiac, brain and placental development that is implicated in HLHS.

## INTRODUCTION

Hypoplastic left heart syndrome (HLHS) is one of the most severe forms of congenital heart disease (CHD), with an estimated prevalence of 1.6 per 10,000 live births (Leirgul et al., 2014). Clinically, it is characterized by the presence of a hypoplastic or absent left ventricle along with a spectrum of underdeveloped left-sided cardiac structures (Franklin et al., 2017). This can include hypoplasia of the ascending aorta and/or aortic arch, and hypoplasia/stenosis/atresia of the mitral and/or aortic valves. Functionally, severe underdevelopment of the left heart results in single-ventricle physiology and this leads to a retrograde pattern of blood flow across the aortic arch (Feinstein et al., 2012). This abnormal circulatory pattern occurs because of a redirection of blood away from the hypoplastic left heart towards the larger right heart. Here, this oxygenated blood mixes with deoxygenated blood before traveling retrograde along the aortic arch, through the patent arterial duct *in utero*, in order to perfuse the coronary and cerebral vasculature.

In HLHS, the cellular and molecular mechanisms leading to left heart growth failure are largely unknown (Grossfeld et al., 2019), and, as a result, development of treatments aimed at ameliorating left heart hypoplasia have been hindered. Indeed, there is currently no cure for HLHS and treatment options typically include cardiac transplantation or aggressive surgical palliation (Feinstein et al., 2012). Although these strategies have made it possible for HLHS newborns to reach adulthood, management of HLHS is resource intensive and, despite its low prevalence, it has been shown to impose the greatest burden in terms of hospitalization costs due to CHD (Simeone et al., 2014). Furthermore, although surgical palliation in HLHS is life sustaining, it is associated with high mortality during childhood (∼60% transplant-free survival to 6 years of age) as well as significant morbidities, including seizures and thromboembolic events (Newburger et al., 2012, 2018).

Apart from these findings, neurodevelopmental disorders remain a major comorbidity in HLHS and other severe types of CHDs (Goldberg et al., 2011). Recent neuroimaging studies have shown a higher risk of brain dysmaturation in this population prior to birth (Clouchoux et al., 2013; Limperopoulos et al., 2010; Sun et al., 2015). Although the mechanisms for disordered brain development in CHDs are not known, converging evidence suggests that circulatory disturbances stemming from structural heart defects, such as retrograde aortic arch flow, likely play an important role, as this circulatory pattern is thought to result in chronic reductions in cerebral oxygenation during critical periods of brain development (Morton et al., 2017a). However, other factors, such as shared genetic defects between heart and brain development, as well as underlying placental disease, may also contribute to the observed neuroimaging phenotypes in a proportion of cases.

The etiology of HLHS and its associated fetal abnormalities is complex. Proposed risk factors include impairments in hemodynamics and intrinsic gene networks (Grossfeld et al., 2019; Morton et al., 2017a). Indeed, the multifactorial etiology of HLHS and its associated fetal abnormalities has made it challenging to determine to what extent and how the hypothesized risk factors drive abnormal heart, brain and overall fetal development. Untangling how intracardiac flow disturbances can act in isolation and in combination with other risk factors to impact cardiac and overall fetal biology will be critical for the development of mechanism-based cardiac and neuroprotective therapies that are urgently lacking. The main difficulty in resolving these questions is the lack of adequate animal models. Indeed, current animal models simulating left heart hypoplasia have limited utility as they either do not fully reproduce the HLHS phenotype (structural and hemodynamic abnormalities), do not survive to term or suffer from high rates of mid-gestational lethality (∼20% survival rate), and/or have low disease penetrance (between 15% and 26%) (Chaturvedi et al., 2019; Fishman et al., 1978; Harh et al., 1973; Liu et al., 2017; Pesevski et al., 2018).

To overcome these significant challenges, we have developed a novel surgical method for producing a mouse model of HLHS that is cardiac specific (and therefore able to isolate the effects of cardiac-induced circulatory disturbances on the fetus from other hypothesized risk factors). Furthermore, experimental mice survive to gestational term and exhibit a fully penetrant isolated HLHS phenotype, which thus far has not been possible. A murine model of HLHS offers several advantages. Mice are widely available to researchers, amenable to surgical procedures and, when combined with advanced imaging technologies (as we have done here to validate our model), allow for tractable and quantitative investigation of the heart following surgical manipulation. Furthermore, mice and humans have homologous heart structures, and the stages of heart development are remarkably conserved (Krishnan et al., 2014). Lastly, compared to large-animal models, mice have a short gestational period and large litter sizes, non-seasonal breeding (compared to sheep, for instance) and are less costly to house. Together, studies in mice – and our model in particular – allow for considerably higher sample sizes, the generation of large datasets and the use of well-established molecular tools to investigate disease mechanisms.

In this study, we combined ultrasound-guided microinjection techniques and biomaterial engineering to deliver an embolizing agent [shear thinning biomaterial (STB)] that is limited to the fetal left atrium following injection. By performing targeted embolizations of the murine left atrium at embryonic day (E)14.5, we blocked the streaming of oxygenated blood flow into the left-sided cardiac structures, inducing severe hypoplasia of the left ventricle, ascending aorta and aortic valve by gestational term (E18.5). Furthermore, by using high-frequency ultrasound biomicroscopy imaging to evaluate aortic arch flow patterns (Color Doppler ultrasound) and *ex vivo* high-resolution three-dimensional magnetic resonance imaging (MRI) for structural phenotyping of the heart and localization of the embolizing agent, we show that our method reproduces the key functional and structural cardiac deficits associated with isolated, single-ventricle HLHS, and at a higher penetrance and survival rate compared to existing models.

## RESULTS

In our study, fetal survival rates at gestational term were 76% for sham fetuses (needle advancement into the left atrium without embolization) and 56% for embolized fetuses (Table 1). From the fetuses that were alive at term in the embolized group, 48% were observed to have positive embolization of the left atrium as determined from MRI (12 out of 25 fetuses). Furthermore, we did not observe any changes in fetal pallor between the experimental groups.

**Table 1.**
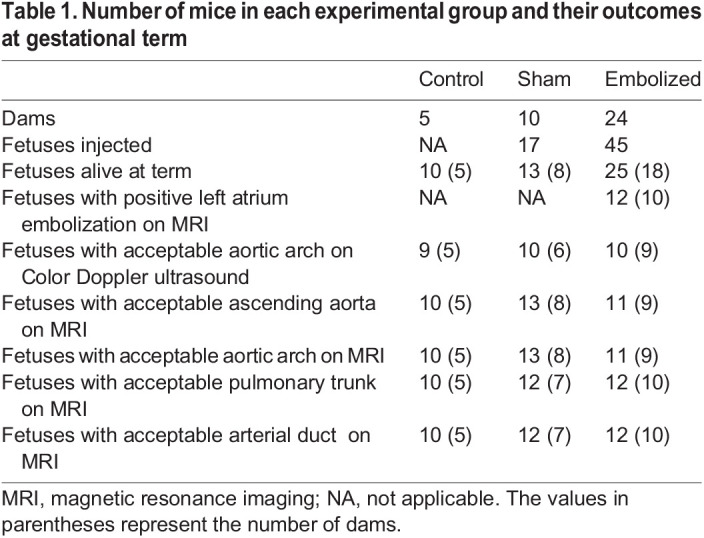
Number of mice in each experimental group and their outcomes at gestational term

For the ultrasound and MRI analysis, all decisions regarding acceptable image quality and data exclusion were made prior to performing any vascular segmentation or statistical analysis. For ultrasound imaging, no aortic arch Color Doppler ultrasound was obtained from one positively embolized and one sham fetus due to imaging time constraints. Furthermore, for one control, one embolized and two sham fetuses, the Color Doppler imaging was retrospectively observed to be inadequate as the imaging view did not contain both the aortic arch and the arterial duct vessels.

For the assessment of aortic valve hypoplasia, the caliber of the aortic and pulmonary valves could not be compared for one embolized, two control and two sham fetuses due to inadequate valvular contrast on MRI. For vascular segmentation, the ascending aorta from one positively embolized fetus (Fig. S1A) and the aortic arch from another positively embolized fetus (Fig. S1B) was not segmented as it contained substantial distal fragmentation of STB as observed on MRI. Additionally, in one sham fetus, the pulmonary trunk and the arterial duct vessels were not segmented due to unclear definition on MRI of the point where the pulmonary trunk divides into right and left pulmonary arteries to become the arterial duct. One fetus from the embolized group was excluded from all analyses as, from visualizing the blood-STB contrast on MRI, it was unclear whether this fetus was positively embolized. Lastly, fetuses in which embolization occurred in the wrong location (12 out of 25 fetuses) were excluded from all analyses.

### Fetuses exposed to the surgical procedure weigh less than controls, independent of the microinjection procedure

**Fig. 1.**
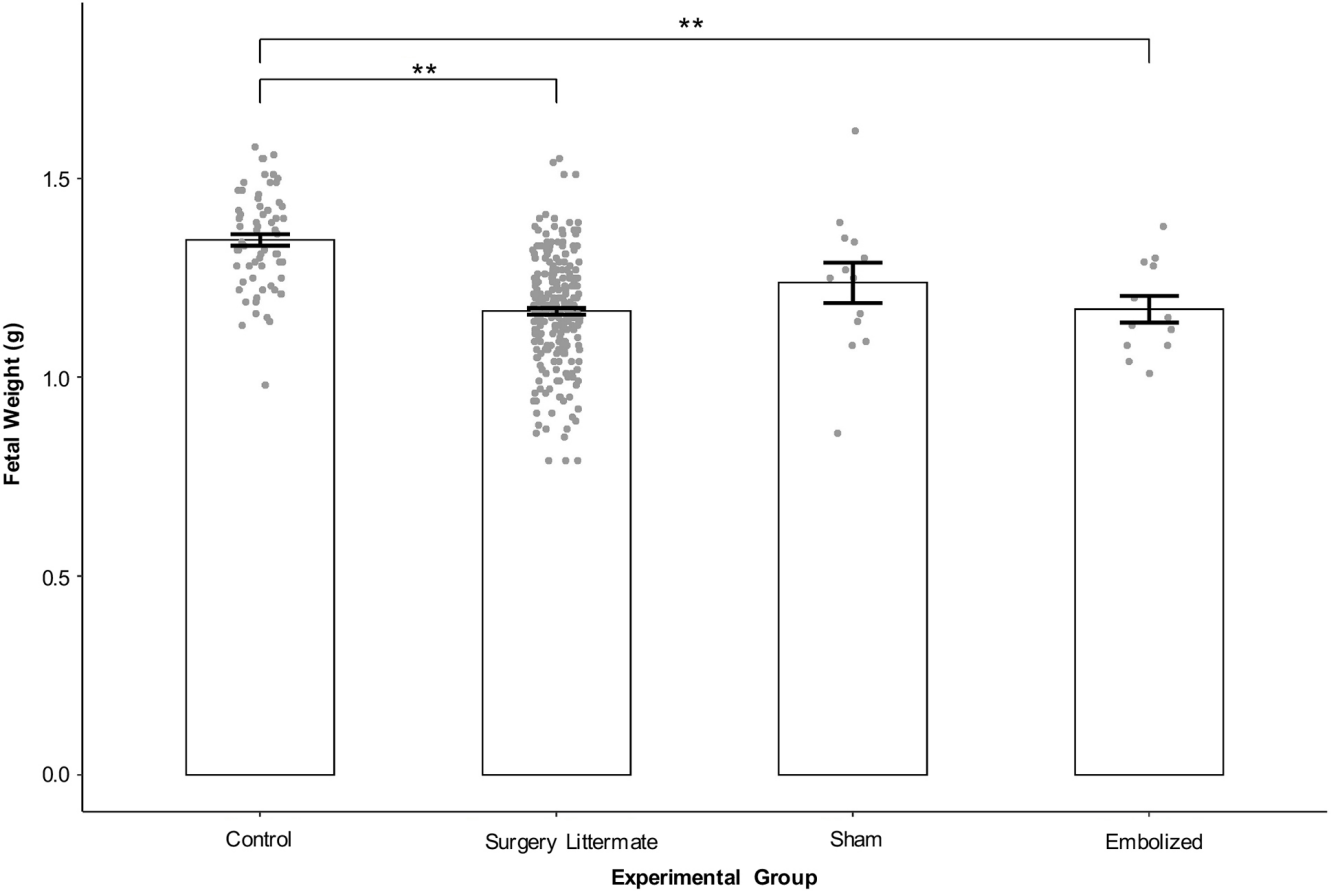
**The effects of surgical procedure and microinjection on fetal weights.** The fetal weights were modeled with a linear mixed effects model (fixed effect, experimental group; random effect, litter). For statistical analysis, a one-way ANOVA was performed on the linear mixed effects model to determine whether there was a group effect. Post-hoc tests were performed by releveling the linear mixed effects model with different experimental groups chosen as the reference group in order to evaluate all possible combinations of between-group comparisons. Data are presented as mean±s.e.m. Control, *n*=67 and *N*=5; surgery littermate, *n*=247 and *N*=26; sham, *n*=13 and *N*=8; embolized, *n*=12 and *N*=10. *N* refers to the number of dams and *n* refers to the number of fetuses. **P<0.01.

A one-way ANOVA showed significant differences in fetal weights between the experimental groups at gestational term (*P*<0.01). In post-hoc analysis (Fig. 1; Table S1), the average weights of surgery littermate fetuses (littermates of sham and embolized fetuses) and the positively embolized fetuses were ∼13% less than those of controls (no surgery performed and pregnancy progressed as normal) (control, 1.35±0.015 g versus surgery littermate, 1.17±0.009 g, *P*<0.01; versus embolized, 1.17±0.034 g, *P*<0.01). Furthermore, the sham fetuses weighed 8% less than controls although this difference was not statistically significant (sham, 1.24±0.051 g, *P*=0.069). There were no statistically significant differences in fetal weights between the surgery littermate, sham and embolized groups. Lastly, inter-litter variation explained 47% of the total variation in fetal weights (inter-litter standard deviation, 9.615×10^−2^ g; intra-litter standard deviation, 1.017×10^−2^ g).

### Positive embolization is associated with retrograde aortic arch flow

**Fig. 2.**
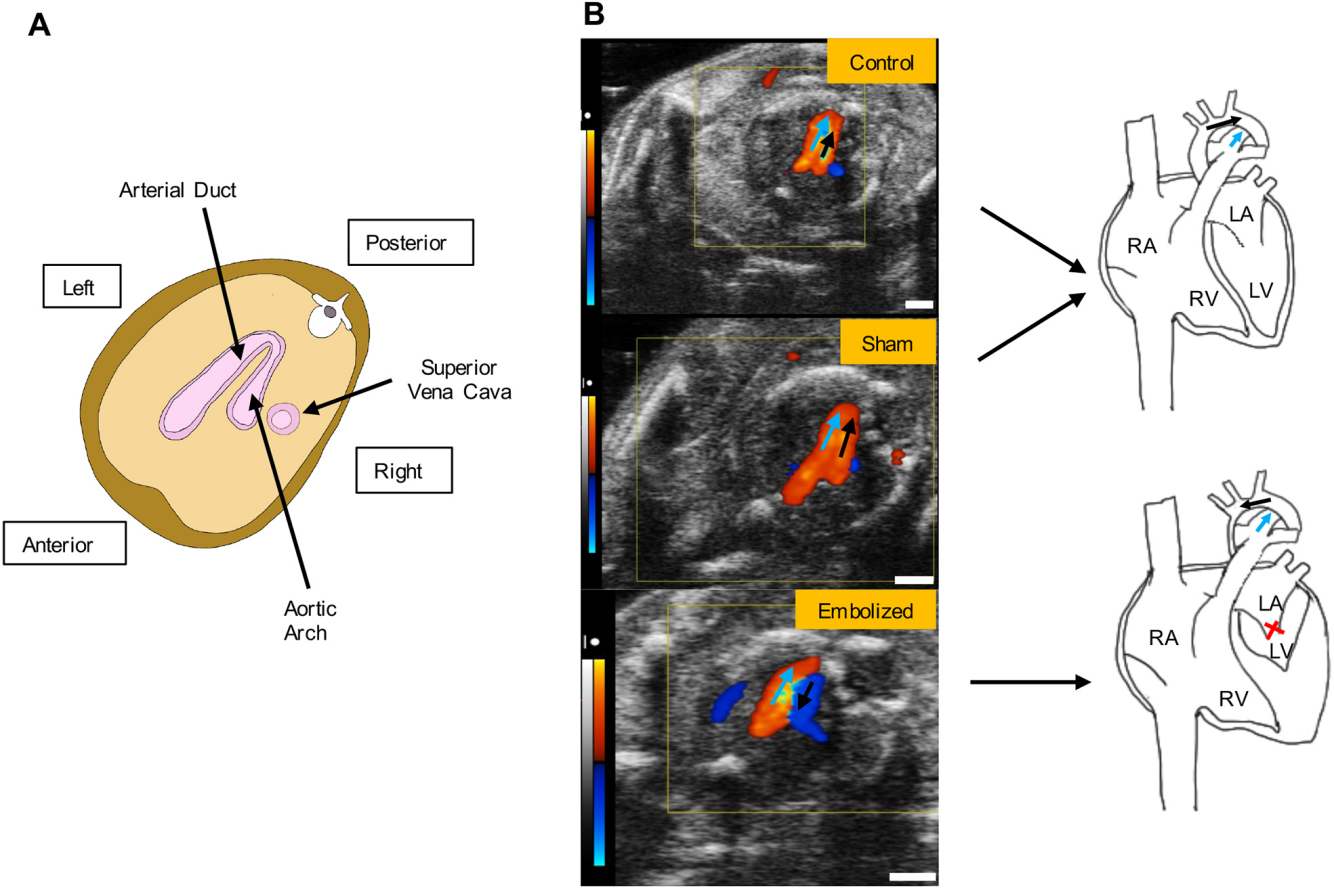
**Aortic arch flow patterns as assessed with Color Doppler ultrasound.** (A) Three-vessel imaging view. (B) Comparison of representative arterial duct (blue arrows) and aortic arch flow (black arrows) patterns in a control (top), sham (middle) and embolized (bottom) fetus at gestational term. On Color Doppler ultrasound images, red indicates flow towards the transducer and blue indicates flow away from the transducer. In control and sham hearts, flow in both the arterial duct and aortic arch vessels is in the same direction; in the embolized heart (where blood flow in to left ventricle was interrupted as indicated by the red cross), flow in the aortic arch and arterial duct vessels is in opposite directions. LA, left atrium; LV, left ventricle; RA, right atrium; RV, right ventricle. Scale bars: 1 mm.

In fetuses with retrograde aortic arch flow (*n*=10 fetuses from nine dams; Fig. 2B), all had positive left atrium embolization as confirmed on MRI. All fetuses with antegrade aortic arch flow (*n*=19) either belonged to the control or sham groups (control, *n*=9 fetuses from five dams; sham, *n*=10 fetuses from six dams). Fisher's exact test showed a significant association between embolization status and aortic arch flow patterns (*P*<0.001; Table S2).

### Positive embolization results in severe left heart hypoplasia

**Fig. 3.**
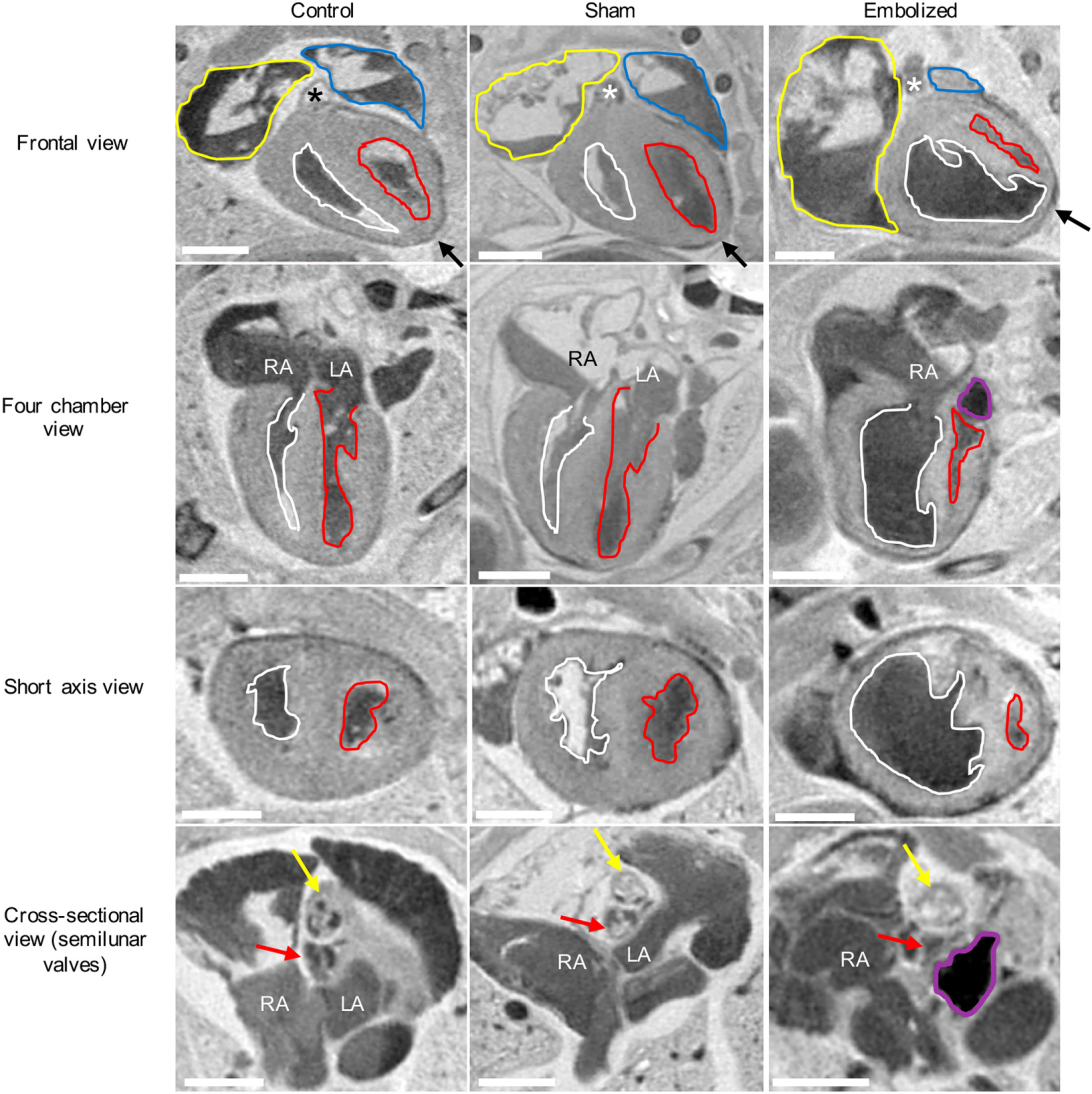
**Two-dimensional MRI slices of the term fetal heart for control, sham and embolized groups.** Compared to the control and sham groups, the embolized group displays severe underdevelopment of the left ventricle and the aortic valve. The yellow outlines indicate the right atrium and appendage, the blue outlines indicate the left atrium and appendage, the white outlines indicate the right ventricle, the red outlines indicate the left ventricle, and the purple outlines indicate the location of the shear thinning biomaterial (STB) embolus inside the left atrium. The asterisks denote the location of the pulmonary trunk, the black arrows indicate the location of the cardiac apex, the yellow arrows indicate the pulmonary valve, and the red arrows indicate the aortic valve. In the first three rows, the cardiac images are from a single mouse for each experimental condition. In the last row, a different mouse was used to highlight the valvular morphology due to superior image contrast. LA, left atrium; RA, right atrium. Scale bars: 1 mm.

**Fig. 4.**
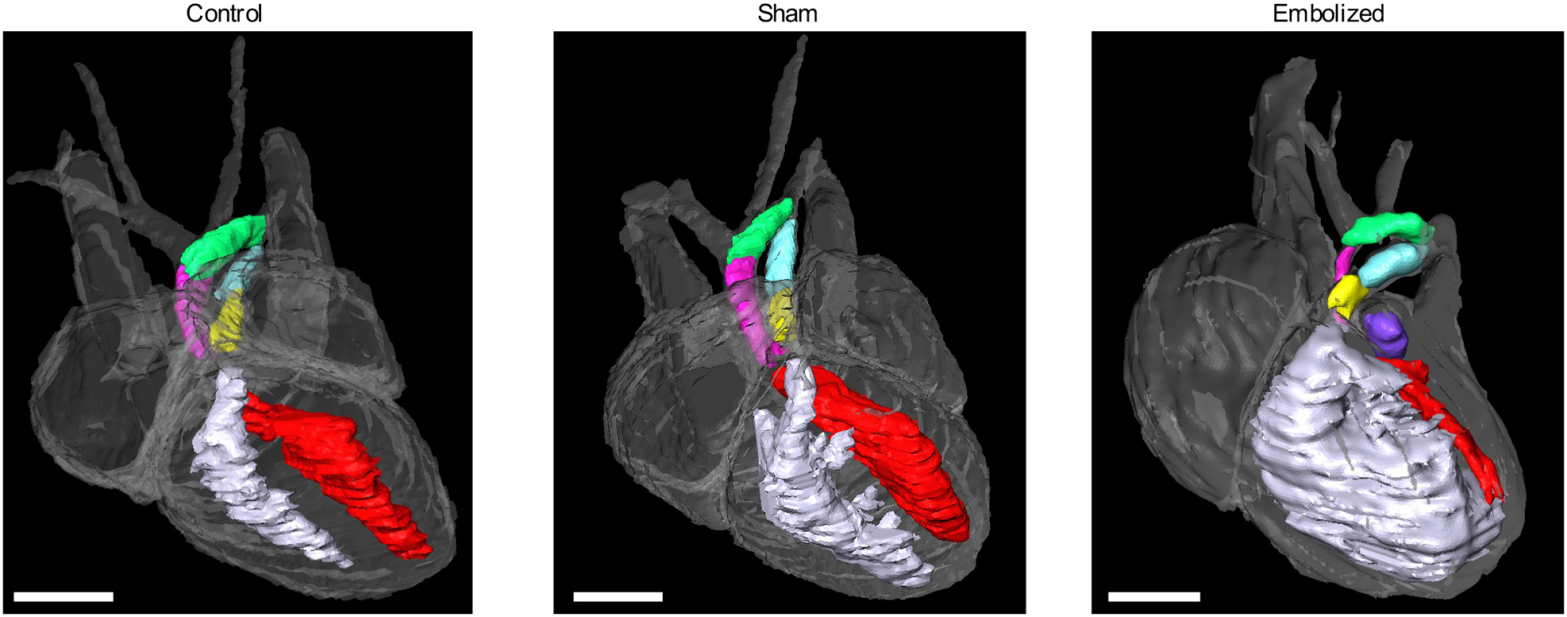
**Three-dimensional volume renderings of term fetal hearts for control, sham and embolized groups.** In control and sham hearts, the left ventricle (red) and right ventricle (white) chambers are similar in size and the cardiac apex is formed by the left ventricle. In the embolized heart, the right ventricle is enlarged and forms the cardiac apex, whereas the left ventricle is hypoplastic and slit-like in morphology. Images were rendered from one representative subject from each experimental condition. Pink, ascending aorta; green, aortic arch; yellow, pulmonary trunk; blue, arterial duct; purple, embolizing agent (STB). Scale bars: 1 mm.

The three-dimensional T2-weighted magnetic resonance images of exsanguinated fetuses provided adequate contrast to discern between cardiac tissue (which appeared light gray), cardiac and vascular lumen (which appeared bright where filled with fluid or dark where blood products were present), and the embolizing agent (which appeared black) (Fig. 3). On MRI, all control and sham fetuses had a cardiac apex formed by the left ventricle, whereas all positively embolized fetuses had a non-apex-forming left ventricle (Figs 3 and 4). Furthermore, the aortic and pulmonary valves were similar in caliber in the control and sham groups. In the embolized group, the aortic valve appeared to be hypoplastic compared to the pulmonary valve in all fetuses except one. In this fetus, the embolizing agent fragmented and occluded a substantial portion of the ascending aorta (Fig. S1A).

**Fig. 5.**
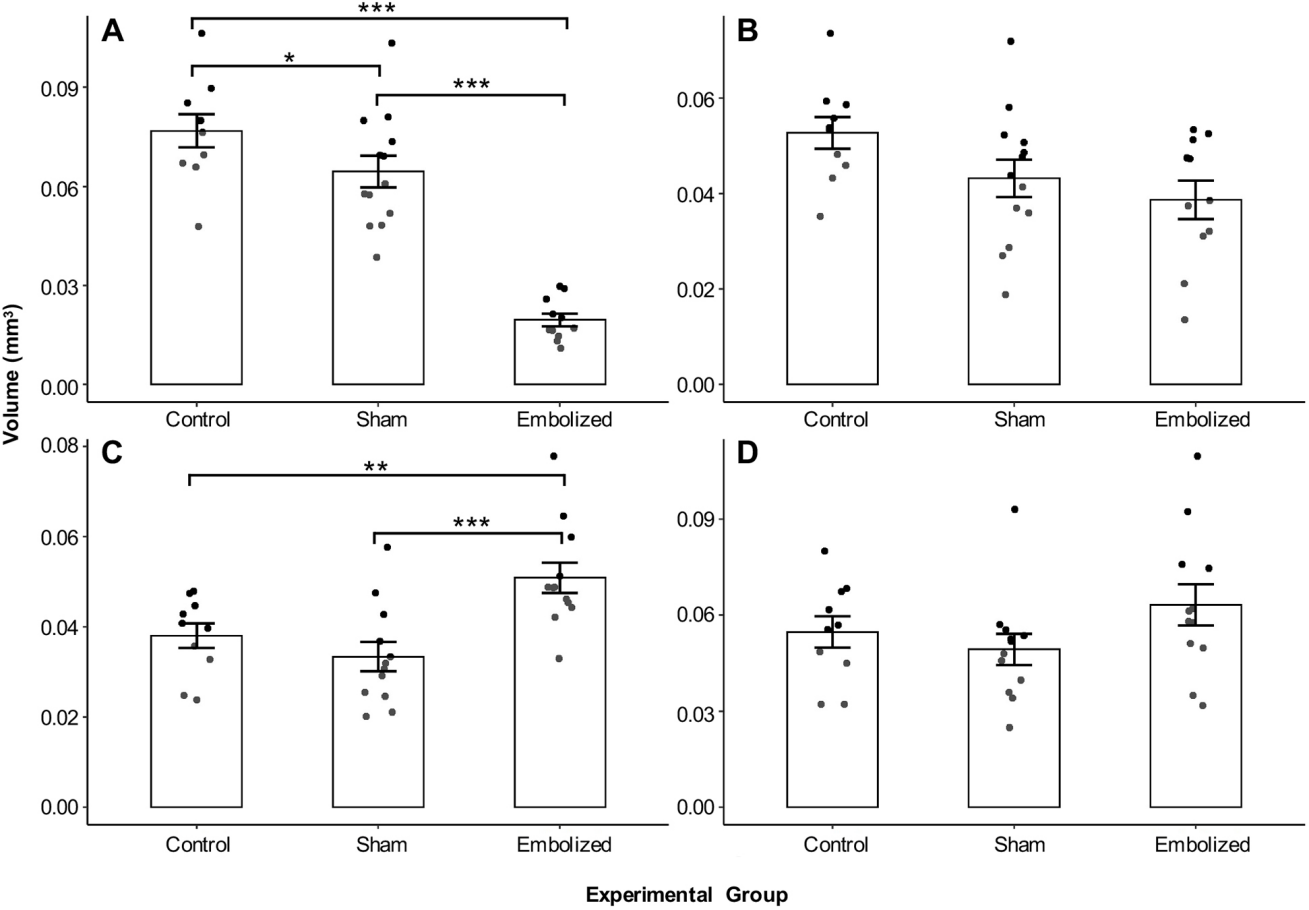
**Vascular volumes derived from magnetic resonance images.** (A) Ascending aorta volume (control, *n*=10; sham, *n*=13; embolized, *n*=11). (B) Aortic arch volume (control, *n*=10; sham, *n*=13; embolized, *n*=11). (C) Pulmonary trunk volume (control, *n*=10; sham, *n*=12; embolized, *n*=12). (D) Arterial duct volume (control, *n*=10; sham, *n*=12; embolized, *n*=12). The vascular volumes were modeled with a linear model (fixed effect, experimental group). For statistical analysis, one-way ANOVAs were performed on the four linear models to determine whether there was a group effect, followed by correction of these four *P*-values for multiple comparisons using the Bonferroni method. Post-hoc tests were performed by releveling the linear model with different experimental groups chosen as the reference group in order to evaluate all possible combinations of between-group comparisons. Data are presented as mean±s.e.m. *n* refers to the number of fetuses. *P<0.05, **P<0.01 and ***P<0.001.

Among the measured vascular volumes (Fig. 5), only the ascending aorta and pulmonary trunk volumes had a significant group effect following correction for multiple comparisons (*P*<0.01). Post-hoc analysis revealed that the average ascending aorta volume was ∼75% smaller in embolized fetuses than in controls (embolized, 1.95×10^−2^±1.91×10^−3^ mm^3^ versus control, 7.68×10^−2^±4.98×10^−3^ mm^3^, *P*<0.001) and 70% smaller than in shams (versus sham, 6.45×10^−2^±4.82×10^−3^ mm^3^, *P*<0.001). Conversely, the average pulmonary trunk volume was ∼34% larger in embolized fetuses than in controls (embolized, 5.09×10^−2^±3.37×10^−3^ mm^3^ versus control, 3.81×10^−2^±2.74×10^−3^ mm^3^, *P*<0.01) and 52% larger than in shams (versus sham, 3.34×10^−2^±3.24×10^−3^ mm^3^, *P*<0.001). Lastly, the average ascending aorta volume was 16% smaller in the sham group than in the control group (*P*<0.05) (Tables S3 and S4).

### Half of positively embolized fetuses show distal fragmentation of STB

From the 12 positively embolized fetuses, six showed fragmentation of STB into the vasculature (Fig. S1). In general, fragmentation was observed in the ostia of vessels branching off the aortic arch or subclavian artery such as in the ostium of the left subclavian artery (one fetus), ostium of the brachiocephalic artery (two fetuses) and ostium of the right axillary artery (one fetus). In two fetuses in which the primary fragment was found in the aortic arch to brachiocephalic artery branching junction, distal fragments were also observed in either the right or left common carotid vessels. Lastly, in two fetuses, the STB fragment was found to occlude the ascending aorta and a portion of the aortic arch.

## DISCUSSION

In this study, we show that targeted embolization of the murine fetal left atrium under high-frequency ultrasound guidance results in a fully penetrant HLHS phenotype at gestational term. The cardiac structural and functional phenotypes were interrogated with high-resolution three-dimensional MRI and Color Doppler ultrasound of the aortic arch, respectively. The former revealed that, compared to the control and sham groups, fetuses with positive left atrium embolization had a non-apex-forming left ventricle, a hypoplastic ascending aorta and aortic valve, and an enlarged pulmonary trunk. This suggests a compensatory enlargement in the right side of the heart to accommodate systemic flow in response to severe left heart obstruction. Furthermore, all positively embolized mice demonstrated retrograde aortic arch flow at gestational term, while all the control and sham mice had antegrade aortic arch flow. Our findings support the flow-volume hypoplasia hypothesis of HLHS (Harh et al., 1973) and indicate that a primary defect in the mitral valves, such as mitral valve atresia/stenosis that would limit the amount of blood flow entering the left ventricle (as simulated by partial blockage of the left atrium in our study), can lead to secondary growth failure of the left ventricle and ascending aorta. Taken together, this indicates that targeted embolization of the left atrium in fetal mice reproduces the key structural and functional cardiac deficits associated with single-ventricle HLHS.

The conventional definition of HLHS requires the presence of normally aligned great vessels and concordant atrioventricular connections, as defined by the Nomenclature Working Group of the International Working Group for Mapping and Coding of Nomenclatures for Paediatric and Congenital Heart Disease (Tchervenkov et al., 2006). Furthermore, this definition typically excludes phenotypes in which a large ventricular septal defect is present along with some degree of left heart hypoplasia. Instead, those cardiac lesions are classified as ‘hypoplastic left heart syndrome-related malformation’ (Tchervenkov et al., 2006). Thus, to recapitulate left heart hypoplasia with the anatomical features as outlined by the Nomenclature Working Group, we performed embolizations at E14.5 in mice, because at this time point the interventricular communication is closed and the normal morphological arrangement of the great vessels, as observed in the post-cardiogenesis heart, has been established (Krishnan et al., 2014; Savolainen et al., 2009; Zhou et al., 2018) (Fig. S5B). Indeed, in our study, all embolized fetal mice at gestational term not only had severe left heart hypoplasia, but also normally aligned great vessels without a common atrioventricular junction and an intact ventricular septum. Similarly, previous morphological studies of post-mortem HLHS specimens have shown that although the left ventricular morphology in HLHS can vary, the arrangement of the great vessels is normal and the ventricular septum is largely found to be intact (Crucean et al., 2017; Stephens et al., 2020). These findings and our own observations suggest that the cardiac lesions observed in HLHS likely represent changes occurring during the post-cardiogenesis period (i.e. following the closure of the interventricular communication) and thus give support to the hypothesis that HLHS can be an acquired disease of fetal life (Anderson et al., 2021). Future studies in which embolizations are performed earlier than E14.5, prior to closure of interventricular communication and proper alignment of the great vessels, will be important for further probing this hypothesis.

As mentioned previously, apart from underdevelopment of the left heart, the definition of HLHS requires the presence of specific anatomical features such as concordant atrioventricular and ventriculoarterial connections. This is an important distinction as a hypoplastic left ventricle can be observed in the context of other CHD phenotypes such as a double-outlet right ventricle or atrioventricular septal defects. Recently, the *Ohia* mouse model was recovered through a large-scale N-ethyl-N-nitrosourea (ENU) mutagenesis screen (Liu et al., 2017). In this model, mutation in the *Sap130* gene was shown to be responsible for left ventricle hypoplasia, while mutation in the *Pcdha9* gene was linked to aortic abnormalities. However, some authors have interpreted the phenotypic findings in the *Ohia* mouse model as an example of a hypoplastic left ventricle rather than hypoplastic left heart syndrome, owing to the presence of anatomical features such as a double-outlet right ventricle in a proportion of these mice (Chaudhry et al., 2019). Furthermore, given the observation of cardiac abnormalities prior to E14.5 in this mouse model, this suggests that the left ventricle hypoplasia in the *Ohia* mouse is likely due to abnormalities occurring during the cardiogenesis period rather than the post-cardiogenesis period, as observed in our mouse model, when major cardiac morphogenesis events (such as looping, closure of the interventricular communication and alignment of the aorta with the left ventricle) are complete and the heart undergoes a period of growth (Krishnan et al., 2014; Savolainen et al., 2009; Zhou et al., 2018). Lastly, although the *Ohia* model supports the idea that a hypoplastic left ventricle phenotype is associated with intrinsic defects, it is unclear whether abnormal blood flow contributes to the structural abnormalities observed in this mouse model.

In the sham group, the ascending aorta volume was, on average, 16% smaller than that of the control group. This reduction was small compared to the severe left heart hypoplasia and reduced aortic volume seen in the positively embolized group. All sham fetuses exhibited antegrade flow with no functional deficit, suggesting that the hypoplasia of the ascending aorta in sham mice was mild and may be explained by their smaller fetal weights.

Furthermore, we found that the fetal weights of embolized mice were smaller than those of the control group. However, the reductions were similar to the decreases observed in fetal weights of the sham and embolized mice's littermates, which encompassed a group that had no advancement of the microinjection needle into the heart. This indicates that the smaller fetal weights observed in the embolized group are likely due to the surgical procedure and manipulation of the uterus, rather than the hemodynamic effects of the HLHS phenotype alone. Similarly, comorbid conditions such as underlying placental disease likely explain the observation of reduced growth in a proportion of HLHS cases (Jones et al., 2015) rather than impaired heart function alone.

In summary, the etiology of HLHS is likely multifactorial, and although *in vitro* studies suggest that intrinsic cardiomyocyte and endocardial defects may contribute to left heart hypoplasia (Miao et al., 2020; Yang et al., 2017), our results indicate that additional factors affecting the hemodynamic loading of the left heart in the fetal period can directly contribute to the pathogenesis of HLHS.

### Advantages of the current method

To our knowledge, we have developed the first cardiac-specific mouse model of HLHS. Previously developed models in the fetal lamb and chick surgically manipulated intracardiac flow patterns to induce various features of HLHS (Chaturvedi et al., 2019; Fishman et al., 1978; Harh et al., 1973; Pesevski et al., 2018). In the early studies of mid-gestational fetal lamb, a balloon catheter was advanced into the left atrium and filled with silicone rubber to block blood flow into the left ventricle. Post-surgical procedure, a decrease in left ventricle growth was observed. However, the average survival time was only 4 days (Fishman et al., 1978). In a more recent study, coil embolization of the fetal lamb left atrium was performed and, at mid-gestation, left ventricle and ascending aorta hypoplasia were observed, with 60% of survivors displaying retrograde flow in the ascending aorta (Chaturvedi et al., 2019). In the chick model, left heart hypoplasia has been induced by placing a nylon suture device across the left atrioventricular canal (Harh et al., 1973) or performing left atrial ligation (Pesevski et al., 2018). In the former method, one out of 32 operated chicks displayed both left ventricle and ascending aorta hypoplasia between E12 and term (E21). The disease penetrance (small left ventricle and ascending aorta/absent ascending aorta) in the chick model utilizing the nylon suture device was only 15% post-surgical procedure (six out of 39 operated embryos). Similarly, in the *Ohia* mouse model, disease penetrance in the double homozygote mutants was 26% (23/88 mutants) (Liu et al., 2017), and a recent study suggests that these mutants suffer from high rates of mid-gestational lethality, likely due to placental defects (Wilson et al., 2021 preprint).

In comparison to these models, our targeted approach results in operated mice surviving to gestational term and exhibiting both structural and hemodynamic abnormalities observed in HLHS at a much higher rate (one out of four operated mice). Furthermore, all positively embolized mice demonstrated retrograde aortic arch flow and underdevelopment of the left heart; thus, the penetrance of HLHS in our model was 100%.

Second, as our mouse model is cardiac specific and displays retrograde aortic arch flow, it is able to assess the direct impact of HLHS-induced circulatory disturbances on fetal development. This will have important implications for understanding how abnormal intracardiac flow patterns lead to left heart growth failure in HLHS and, more broadly, how circulatory disturbances due to a severe CHD can directly impact fetal biology. For instance, owing to the lack of viable animal models, recent attempts to investigate the cardiac–neurodevelopmental axis in severe CHD have involved exposing model organisms to hypoxia in order to mimic the circulatory component that is thought to result in decreased cerebral oxygenation and brain dysmaturation across various types of CHDs (Morton et al., 2017b). Furthermore, the *Ohia* mouse model of left ventricle hypoplasia displays mutations in genes that are expressed widely in the embryo (Liu et al., 2017), including the brain, thus precluding the investigation of how circulatory patterns in CHD directly lead to abnormal brain and overall fetal development. In comparison, our model is well positioned to address the direct impact of CHD-induced circulatory disturbances on fetal biology, such as neurodevelopment, cardiac development and placental development, all of which have been implicated in CHD.

Lastly, when combined with genetically engineered mouse models of placental disease or genetic abnormality of interest in the heart or brain, our method of surgically inducing HLHS may also reveal the combined contributions of genetic defects, placental disease and fetal hemodynamics on the developing HLHS fetus. This is particularly advantageous because modeling severe CHD in the mouse through genetic modification often results in perinatal lethality/widespread disturbances in other organs (Rufaihah et al., 2021). Lastly, our technique can be used to induce HLHS at earlier or later time points of gestation to modulate disease severity and it has the potential to be adapted to larger animal models.

### Limitations

The fetal survival rate in our study was 56% for the embolized group and 76% for the sham group. These survival rates are within the range observed in previous microinjection studies. For instance, Slevin et al. (2006) observed a survival rate of 56% at birth following sham surgeries conduced at E7.5 (surgical procedure but without the microinjection needle advancement). However, based on our experience, the fetal survival rates in the embolized group can be improved by minimizing the angle of the needle while inside the fetal thoracic cavity in order to reach the left atrium target and avoiding piercing of vessels inside the body wall/thoracic cage. In ten fetuses in the embolized group in which these criteria could not be met, we found pronounced bleeding from the site of puncture at E14.5, and, at E18.5, seven of these fetuses were reabsorbed. To avoid this complication, we recommend that if upon needle advancement the tip of the needle is too high or too low for the left atrium target, the needle should be fully retracted (out of the uterus) and the vertical position of the needle should be adjusted before attempting to embolize, rather than angling the needle inside the thoracic cavity. Furthermore, if the needle and the body wall/thoracic vessels (observed as bright moving speckles on B-mode imaging) are encountered in the same imaging plane as the needle, the micro-positioning controls of the animal platform should be adjusted to bring the blood vessels out of the imaging plane before advancing the needle forward.

Of the mice that survived, the positive embolization rate in our study was 48%. In 12 out of 25 fetuses, in which the embolization was not positive on MRI, the STB embolus was observed to be outside of the heart in eight fetuses. During the microinjection procedure, we sometimes encountered slight movement of the left atrium target or the needle out of the imaging plane when advancing the needle through the ribcage (Fig. S2). To improve targeting accuracy, we recommend adjusting the micro-positioning controls on the injector mount and the animal platform to bring the left atrium target and microinjection needle in the same imaging plane once the needle tip has been advanced beyond the ribcage, and injecting the embolus only if the top and bottom walls of the left atrium can be seen surrounding the needle tip.

Furthermore, we found that, in 50% of cases, the STB embolus fragmented distally into the vasculature. This may be due to variance in fetal heart rates, where higher physiological heart rates may result in a greater likelihood of embolus fragmentation. Although STB is a hemostatic agent, the formation of blood clots on the surface of STB (which minimizes the risk of fragmentation) occurs within 3-5 min post-injection (Avery et al., 2016). Lowering fetal heart rates through administration of adenosine (Koeppen et al., 2009) or by transiently increasing the isoflurane anesthesia concentration during this 3-5 min window may reduce the chances of STB fragmentation. Lastly, the method developed here to produce a model of HLHS is technically demanding and we recommend at least 1 month of training for a learner who does not have any experience in performing survival surgeries, ultrasound imaging and microinjection.

In summary, we have presented a novel method to surgically induce HLHS in fetal mice that is validated using ultrasound and MRI. Our approach results in mice surviving to term and displaying a fully penetrant isolated HLHS phenotype, which thus far has not been possible. The development of this model in the mouse will pave the way for investigating the mechanism and evolution of HLHS *in utero*, as well as identifying the mechanisms of fetal abnormalities observed in severe CHDs and testing of preclinical therapies, which has been hindered due to the limited availability of clinically relevant animal models.

## MATERIALS AND METHODS

### Mice

Female virgin CD-1 mice (6-9 weeks of age) were obtained from Charles Rivers Laboratories (St. Constant, QC, Canada) and mated in-house. The morning that a copulation plug was detected was designated as E0.5 (gestational term=E18.5). Three groups of healthy, pregnant CD-1 mice were randomly assigned as follows: control (no surgical intervention and pregnancy progressed as normal, *n*=10 fetuses from five pregnant dams), sham (microinjection needle was advanced into the left atrium but without the embolization, *n*=17 fetuses from ten pregnant dams) and embolized (microinjection needle was advanced into the left atrium and embolizing agent was delivered, *n*=45 fetuses from 24 pregnant dams). For the sham and embolized groups, the surgical procedure was performed at E14.5; the cardiovascular phenotyping was performed at E18.5 for all groups. The surgical procedures for all groups of mice were completed over a period of ∼6 weeks, while alternating between surgical procedures. All animal experiments were approved by the Animal Care Committee (ACC) at The Centre for Phenogenomics (TCP), under Animal Use Protocol 21-0249H, which adheres to the Policies and Guidelines of the Canadian Council on Animal Care and meets all the requirements of the Provincial Statute of Ontario, Animals for Research Act as well as those of the Canadian Federal Health of Animals Act.

### Surgical protocol

#### Embolizing agent

The embolizing agent that was used in the surgical procedure is known as STB. STB is a type of injectable hydrogel that is composed of Type A porcine gelatin (G1890, Sigma-Aldrich) and silicate nanoplatelets (Laponite-XLG XR, ECKART) (Gaharwar et al., 2014). STB has recently been used to embolize the femoral artery of adult mice, where the cast did not fragment and had minimal inflammatory effects (Avery et al., 2016).

In this study, the STB was formulated to have a total solid (gelatin and nanoplatelet) concentration of 6% (w/w), with 75% (w/w) of the total solid composed of silicate nanoplatelets (Avery et al., 2016; Gaharwar et al., 2014). Briefly, a 9% (w/w) silicate nanoplatelet stock was prepared by vortexing Laponite (Laponite-XLG XR, ECKART) into 2°C double-distilled water (ddH_2_O) until a thick gel formed. This took ∼5-7 min, after which the nanoplatelet gel was allowed to rest until it reached room temperature. Next, an 18% (w/w) gelatin (G1890, Sigma-Aldrich) stock prepared in 40°C ddH_2_O was added to the silicate nanoplatelet gel, along with additional ddH_2_O to achieve the 6% solid mass and 94% water mass ratio. This mixture was vortexed for 5 min, then kept in a 40°C warm water bath overnight to achieve an even mixture of gelatin and Laponite particles, and finally vortexed again for 5 min. Using a spatula, the prepared STB gel was backfilled into 1 ml syringes with 26-gauge needle attachments (Becton Dickinson) and then sterilized through γ-irradiation (25 kGy total dose, Gamma Cell, type G.C. 220 irradiator, University of Toronto). The sterilized, STB-filled syringes were stored at 4°C for up to 3 months.

#### Microinjection needle preparation, priming and alignment

Glass microcapillary tubes (3-000-203-G/X, Drummond Scientific; inner diameter, 0.53 mm; outer diameter, 1.14 mm) were pulled using a micropipette puller (Sutter Instrument P-1000) with the following settings: heat, 585 units; pull, 50 units; velocity, 150 units; time, 50 units; pressure, 500 units. This created a ∼15 mm long taper, which was broken to an outer diameter of ∼50 µm. The broken needle tip was beveled to an angle of 20° (in 5° increments starting from 30°) on a moistened grinder for 7 min per angle (Narishige EG-401 Microgrinder). Lastly, the side of the needle barrel that coincided with the beveled edge of the needle tip was labeled with a marker under the Microgrinder microscope.

Prior to the surgical procedure, the STB was back-filled into beveled microinjection needles and inserted into a VisualSonics Vevo Micro-Injection Unit (VisualSonics Image-Guided Injection System), ensuring that the beveled edge of the needle pointed up. To ensure that the microinjection needle was fully primed, and to avoid air bubbles, the STB (contained within 1 ml syringes with 26-gauge needle attachments) was back-filled until it could be seen extruding from the microinjection needle tip. At this point, the 26-gauge needle was slowly retracted from the barrel end while still injecting. This ensured that the microinjection needle was fully primed, from the barrel end to the tip. To further avoid air bubbles, we back-filled with a slow steady pressure. Small air bubbles that form near the microinjection needle tip can be injected out with the microinjection system. It is ideal to avoid air bubbles as they can result in inconsistent injectate volume delivery.

**Fig. 6.**
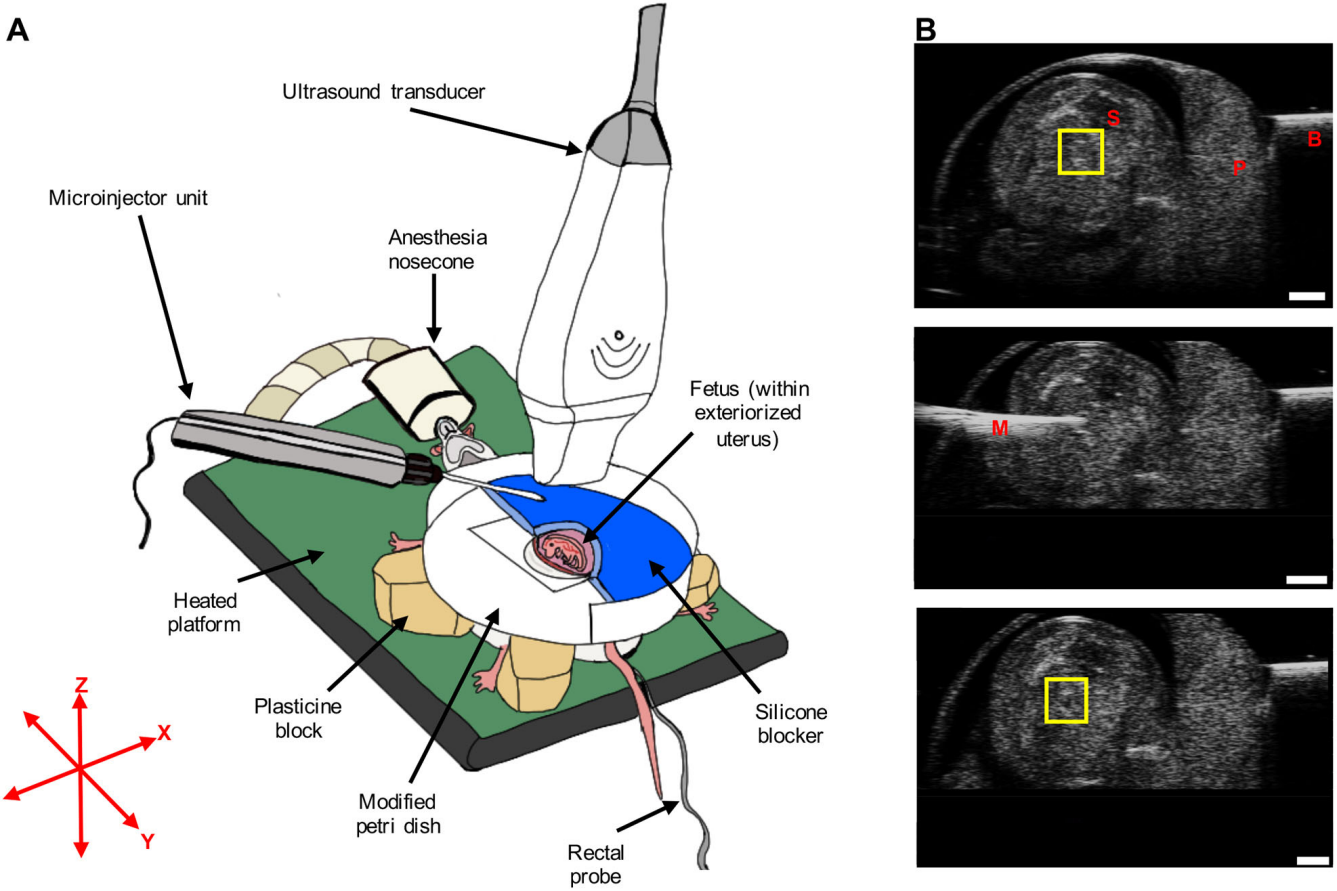
**Surgical procedure and microinjection.** (A) Surgical procedure for ultrasound-guided microinjection into the fetal left atrium. (B) Pre-injection B-mode image (top), microinjection needle advancement B-mode image (middle) and post-injection B-mode image (bottom) from a single representative fetus in the embolized group. In the top panel, the yellow square highlights the region showing the top and bottom walls of the left atrium as bright, horizontal lines. Following injection, a dark spot can be seen in between the top and bottom walls of the left atrium, confirming that embolization was successful (see yellow square in the bottom panel). B, blocker; M, microinjection glass needle; P, placenta; S, spine. Scale bars: 1 mm.

Next, the transducer was fixed in position so that the ultrasound beam was parallel to the XZ plane (Fig. 6A). The filled microinjection needle was aligned under the transducer by using the injection angle control and Y (side-to-side shift) micro-positioning control on the injector mount (Fig. S3A), until the needle was horizontal (parallel to the *x*-axis) and appeared the brightest on ultrasound B-mode imaging.

For the duration of the surgery, the microinjection needle tip was kept moist by keeping the needle tip submerged in phosphate-buffered saline (PBS) in between microinjections (Fig. S3B). This was essential to prevent blockage of the needle tip due to hardening of the embolizing agent when exposed to air for prolonged periods of time. Lastly, prior to performing microinjections, the patency of the needle tip was assessed by visualizing an echogenic stream extruding from the needle on B-mode ultrasound imaging upon microinjection into sterile ultrasound gel.

#### Animal preparation

Pregnant dams at E14.5 were given 2 mg/kg Meloxicam (Metacam, Boehringer Ingelheim) subcutaneously as pre-emptive analgesia at least 30 min prior to surgery, and hair from the abdominal region was removed using depilatory cream (Nair, Church and Dwight). During ultrasound imaging, pregnant mice were anesthetized with 2.5% isoflurane in 100% O_2_ (induction at 4% isoflurane in 100% O_2_) and laid supine on a temperature-regulated platform with paws taped to the electrocardiogram electrodes for monitoring of maternal heart rate. The maternal body temperature was monitored using a rectal temperature probe. During the surgery, both of these physiological parameters were examined every 10 min. Across all dams, the average maternal heart rate and body temperature ranged between 492 and 635 beats/min and 34.6°C and 36.8°C, respectively. Lastly, an ophthalmic ointment (Tears Naturale P.M.) was applied to prevent corneal drying.

#### Surgical procedure

Following a toe pinch test to ensure a deep plane of anesthesia, a 2-3 cm ventral midline incision was made to open the peritoneum and the uterine horn was exteriorized onto the maternal abdominal surface (Fig. 6A). After a visual inspection, the position of fetuses with the best orientation for microinjection was recorded. The criteria for optimal orientation were as follows: (1) the fetus was prone and (2) its left lateral side faced the microinjection needle with the placenta located away from the path of injection. A single conceptus with this orientation was exposed above the incision site, and the rest of the uterine horn was placed back inside the abdominal cavity.

Next, a Petri dish containing a 1 in hole in the middle, covered with a transparent rubber membrane that contained an ∼0.8 cm by 1.4 cm oval-shaped slit for extraction was placed just above the exteriorized uterus. The Petri dish wall, corresponding to approximately half the circumference of the Petri dish, was removed to allow for advancement of the microinjection needle in a horizontal position (Fig. S4). Next, forceps were passed through the oval-shaped slit, and the edges were opposed far enough to encompass the entire conceptus. The fetus within the intact uterine horn was extracted through the opposed slit by advancing the modified Petri dish towards the maternal skin, at which point the forceps were retracted. This created a seal between the maternal skin and the conceptus, separated by the rubber membrane.

Next, the modified Petri dish was stabilized on plasticine blocks. To avoid movement of the conceptus with advancement of the microinjection needle, the uterus was stabilized by placing a half-moon shaped silicone blocker that was flush against the uterus and the wall of the Petri dish. The silicone blocker was made by creating a cast in the shape of the Petri dish (Smooth-Sil™ 945, Smooth-On Inc.). The conceptus was then covered with room temperature sterile ultrasound gel (Sterile Ultrasound Gel USG20-ST, Cardinal Health Canada), which cooled the fetus to lower the heart rate for microinjection and also acted as a coupling medium for ultrasound.

To ensure that the left atrium was within the same imaging plane as the needle tip, the left-right body axis of the prone fetus was tilted (with a cotton swab) until the fetal spine was ∼30-45° clockwise in the XZ plane and the top and bottom walls of the left atrium appeared as bright, horizontal echogenic lines on B-mode ultrasound (Fig. 6B; Movie 1). The microinjection needle was then advanced forward along the *x*-axis until the needle tip was situated centrally within the left atrium in close proximity to the atrial septum and the bright walls of the left atrium could be visualized surrounding the needle tip (Movie 2). Between 50.6 nl and 69.0 nl of the embolization product was delivered (at a rate of 46 nl/s), and embolization was confirmed by observing a hypoechogenic mass within the left atrium cavity on B-mode ultrasound (Movie 3). In a pilot study undertaken to optimize the surgical protocol, the STB material was mixed with food coloring dye and microinjected into left atrium under ultrasound guidance, followed by dissection of fetal hearts (Fig. S5A). Localization of STB revealed this range of injectate volume to be sufficient for occluding the left atrium without causing occlusion of surrounding structures (e.g. left atrial appendage, pulmonary vein and the left ventricle chamber).

During needle advancement, if the fetal limbs and vitelline vessels (Movie 3) were encountered when advancing the needle, the Y micro-positioning control on the injector mount (Fig. S3A) was used to move the needle out of the imaging plane (needle tip appeared less bright), and the needle was then extended forward along the *x*-axis until the needle tip was beyond these targets. Avoiding these structures was critical to avoid hemorrhaging from the limbs and fetal mortality. Prior to microinjection into the left atrium, the needle tip and the left atrium walls were brought back into the same imaging plane by either manipulating the micro-positioning controls of the injector mount and/or the animal platform.

Following confirmation of embolization on ultrasound imaging, sterile gel was removed by washing the conceptus with warm PBS (37°C), after which the uterus was placed back inside the abdominal cavity, such that the original configuration of the uterus was preserved as much as possible (in order to avoid torsion of the uterine vessels). Time permitting, this procedure was repeated for a second/third fetus. Lastly, the maternal muscle and skin layers were closed with 5-0 absorbable sutures (Polysorb Braided Absorbable Suture, Covidien) and wound clips (9 mm AutoClips, MikRon Precision), respectively. The total anesthesia time was kept below 40 min to prevent fetal mortality due to a prolonged surgical procedure (Slevin et al., 2006). Following the procedure, the pregnant dam was allowed to recover in a sterile, prewarmed chamber. All pregnant dams were fully alert and explored the chamber within 4 min post-surgery. Lastly, all materials used during the surgery were sterilized (either through autoclaving or exposure to vaporized hydrogen peroxide) and aseptic survival surgeries were performed according to the ACC guidelines at TCP.

### Phenotyping

#### Ultrasound biomicroscopy

Fetuses were imaged using a high-frequency (40 MHz linear array transducer) ultrasound biomicroscopy system (Vevo 2100, VisualSonics) for both the surgical procedure at E14.5 and cardiovascular phenotyping at E18.5. For the phenotyping, pregnant dams at gestational term were prepared for surgery and ultrasound imaging as described in the previous section, and the conceptuses that were injected at E14.5, as well as control conceptuses, were exteriorized through a ventral midline incision, one at a time. For the control group, two to three conceptuses were randomly selected in each dam. Exteriorization of the uterus at E18.5 was performed because *in vivo* it was difficult to count the position of each conceptus along the uterine horn to locate the fetus that had the microinjection procedure performed at E14.5. This is because the term uterine horn in its normal configuration weaves through the abdominal cavity, going in and out of the imaging plane on two-dimensional B-mode imaging. Furthermore, exteriorization made it possible to orient the fetus appropriately for aortic arch imaging, and it allowed us to image fetuses that were positioned underneath the surgical incision site (which can cause a shadowing artifact due to the presence of scar tissue).

Following exteriorization, the injected fetuses were assessed for survival at E18.5 by determining whether the fetus was reabsorbed or not. If the fetus was not reabsorbed, a B-mode ultrasound scan of the heart was performed to look for contraction to confirm survival. To visualize the direction of blood flow in the aortic arch at E18.5, a four-chamber cardiac view was obtained in Color Doppler mode (Zhou et al., 2018), and the imaging plane was then moved cranially to obtain a transverse view of the aortic arch and the arterial duct as these vessels joined together at the level of the descending aorta (Fig. 2A).

#### MRI

Immediately following ultrasound phenotyping at E18.5, pregnant dams were sacrificed by cervical dislocation and the conceptuses were excised into warm PBS (37°C). Next, the extra-embryonic tissues were removed and the combined fetus, umbilical cord and placental weight was recorded for fetuses that underwent ultrasound imaging. To obtain magnetic resonance images of these fetuses, the umbilical cord (with the attached placental unit) was cut to drain the blood in order to obtain sufficient contrast between the injected STB and blood-filled compartments (both of which appear dark on T2-weighted MRI). To obtain the non-exsanguinated fetal weight, the umbilical-placental weight was subtracted from the combined fetal, umbilical cord and placental weight. These fetuses were then immersion-fixed for MRI in 4% paraformaldehyde and 2 mM ProHance (gadolinium contrast agent, Bracco Diagnostics) for 5 days at 4°C (the fetal skin was removed in several places to aid in penetration of the fixative). Following fixation, fetal samples were placed in a solution of PBS, 2 mM ProHance and 0.02% sodium azide for long-term storage. For the rest of the littermate fetuses that did not undergo MRI, fetal weights were immediately obtained after excision of the umbilical-placental unit.

For anatomical scans, fixed samples were placed in a proton-free susceptibility-matching fluid (Fluorinert FC-77, 3M Corp.) and imaged using a custom-built solenoid array to scan 16 samples in parallel (Dazai et al., 2011). A three-dimensional, high-resolution (40 µm isotropic) T2-weighted fast spin-echo sequence using a cylindrical *k*-space acquisition was performed on a 7.0 Tesla (T) MRI scanner (Varian, Palo Alto, CA, USA) as described previously (Spencer Noakes et al., 2017). The imaging sequence parameters were as follows: repetition time 350 ms, echo time 12 ms; echo train length, 6; four averages; field-of-view, 20 mm×20 mm×25 mm; matrix size, 504×504×630. Samples were imaged with MRI after a period of at least 30 days, as fixative associated tissue volume changes (expansion or shrinkage) after this period largely stabilize (De Guzman et al., 2016).

To evaluate left ventricle growth, three-dimensional magnetic resonance images were qualitatively assessed to determine whether the cardiac apex was formed by the left ventricle. To determine whether the aortic valves were hypoplastic or not, magnetic resonance images were oriented to obtain a cross-sectional view of the aortic and pulmonary valves. Next, the caliber of the aortic valve was qualitatively compared to the caliber of the pulmonary valve for each fetus. To assess the degree of vascular hypoplasia, the ascending aorta, aortic arch, pulmonary trunk and arterial duct vessel volumes were obtained by manually segmenting these vessels in Display (MINC toolkit, McConnell Brain Imaging Centre, Montreal, QC, Canada) and assigning labels. Next, the total number of voxels in each label was counted and multiplied by the individual voxel volume using the anatGetAll() function in the RMINC package (https://github.com/Mouse-Imaging-Centre/RMINC).

The vascular segments were anatomically defined as follows: (1) ascending aorta: the region between the sinutubular junction to just prior to the ostium of the brachiocephalic artery branch; (2) aortic arch: the region extending between the ostium of the brachiocephalic artery to subsequent to the ostium of the left subclavian artery branch; (3) pulmonary trunk: the region between the sinutubular junction and the division of the pulmonary trunk into left and right pulmonary arteries; and (4) arterial duct: the region connecting the pulmonary trunk to the aortic arch by extending from the division of the pulmonary trunk to just after the ostium of the left subclavian artery. Lastly, the fetal heart was qualitatively assessed for confirmation of embolization while the vasculature was assessed for any signs of distal embolization.

### Statistics

Sample size was determined *a priori* based on a power analysis for one-way ANOVA (pwr::pwr.anova.test in R). At an alpha of 0.05, we determined that ten fetuses per group would be sufficient with a power of 0.8 to detect a large effect size of 0.6. Anticipating that 25% of the embolization procedures would be successful (positive embolization and fetal survival) and meet the imaging data quality criteria, we increased the number of litters required proportionally. Owing to the invasive nature of the surgical procedure and large changes observed in the cardiac phenotype, the investigators were not blinded to the group allocation of animals during experiments and at the data analysis stage.

To account for variation in fetal weights, a linear mixed effects model was fitted with litter as a random effect and experimental group as a fixed effect (Bates et al., 2015; Faraway, 2016). Apart from including the weights of control, sham and positively embolized fetuses in the experimental group, we also included the weights of fetuses that were littermates of sham and embolized fetuses (denoted as surgery littermates). We included this group to determine whether the surgical procedure (without the microinjection needle advancement or left atrium embolization) had an effect on fetal weights.

The primary outcome measure was the presence of retrograde flow along the aortic arch in positively embolized fetuses. To determine this relationship, a Fisher's exact test was performed comparing the aortic arch flow patterns (antegrade or retrograde) and embolization status [positive, embolus visualized in left atrium on MRI; negative, no embolization performed (control and sham fetuses)].

To account for variation in vascular volumes, a linear model with experimental group as a fixed effect was fitted for each of the four vessels. For both the linear mixed effects model and the four linear models, a one-way ANOVA was performed to determine whether there was a group effect. We corrected these four *P*-values for multiple comparisons using the Bonferroni method, as implemented in the p.adjust() function in R. If the one-way ANOVA was significant, post-hoc tests were performed by releveling the models with different experimental groups chosen as the reference group in order to evaluate all possible combinations of between-group comparisons. All statistical tests were performed in R statistical software (www.r-project.org). *P*<0.05 was considered significant. All data are presented as mean±s.e.m., with distributions of individual observations overlaid where applicable. *N* refers to the number of dams and *n* refers to the number of fetuses.

## Supplementary Material

Supplementary information
