# Supplementary material for: A mouse model of hypoplastic left heart syndrome demonstrating left heart hypoplasia and retrograde aortic arch flow
Source: Dis Model Mech. 2021 Nov 10;14(11):dmm049077. doi: 10.1242/dmm.049077 (PMC8592017; doi:10.1242/dmm.049077)
Supplement: Supplementary information [file dmm-14-049077-s1.pdf]

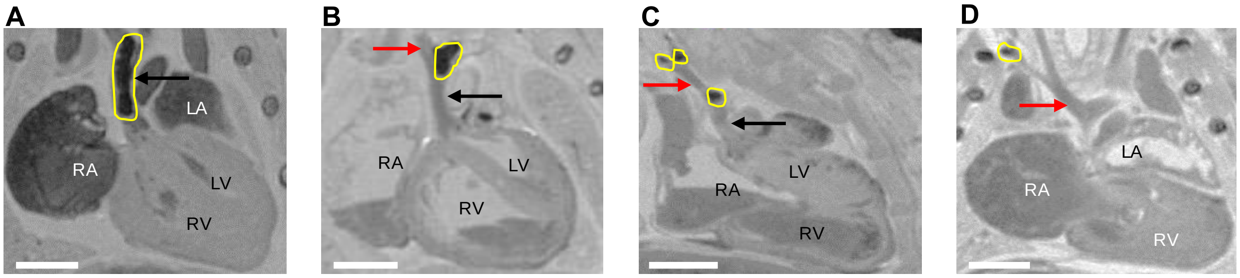

Fig. S1. Distal fragmentation of STB into the vasculature on MRI. In A, the STB fragment was found to fully occlude the ascending aorta. In B, the STB fragment was observed extending from the ostium of the brachiocephalic artery towards the ostium of the left common carotid artery, occluding approximately 50% of the aortic arch. In C, the STB fragment was noticed in the ostium of the brachiocephalic artery and further upstream into the ostia of the right subclavian and right common carotid arteries. In D, an isolated small fragment of STB was noticed in the ostia of the right axillary artery. Yellow outline indicates the location of the STB fragment. Black arrow refers to the ascending aorta and red arrow refers to the brachiocephalic artery. RA: right atrium, LA: left atrium, RV: right ventricle, and LV: left ventricle. Scale bar: 1 mm.

Disease Models & Mechanisms • Supplementary information

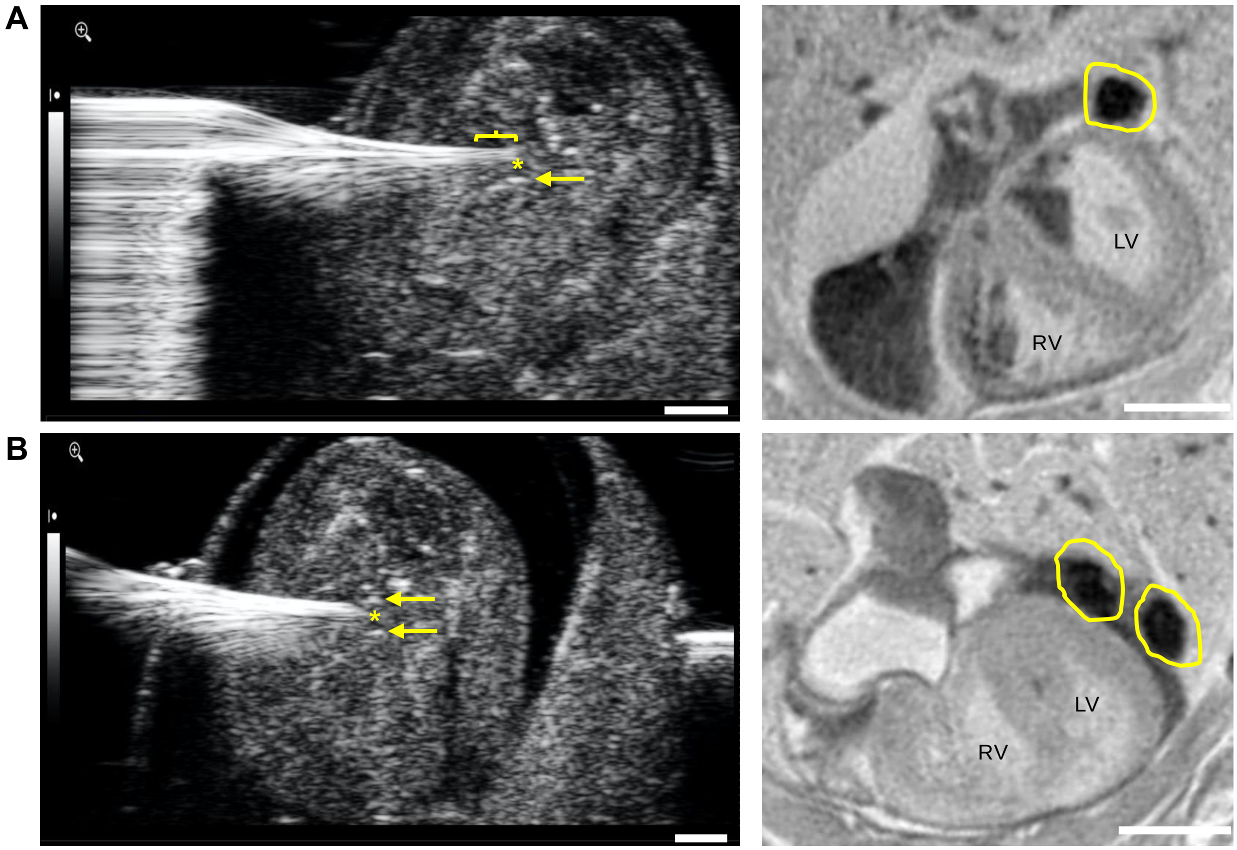

Fig. S2. Factors responsible for inaccurate embolizations. In A, the microinjection needle tip end is out of the B-mode ultrasound imaging plane as its signal intensity drops off (yellow curly bracket) as compared to the rest of the needle which appears bright. In addition, the position of the needle tip is high and located above the left atrium target, which is indicated by the yellow star, and only the bottom wall of the left atrium is visible (yellow arrow) rather than both walls. In B, the needle tip is bright and both the top and bottom walls of left atrium can be visualized. However, the needle tip falls short of the left atrium target. In both A and B, the STB embolus (circled in yellow, right panel) was observed to be outside the heart on MRI. RV: right ventricle and LV: left ventricle. Scale bar: 1 mm.

Disease Models & Mechanisms • Supplementary information

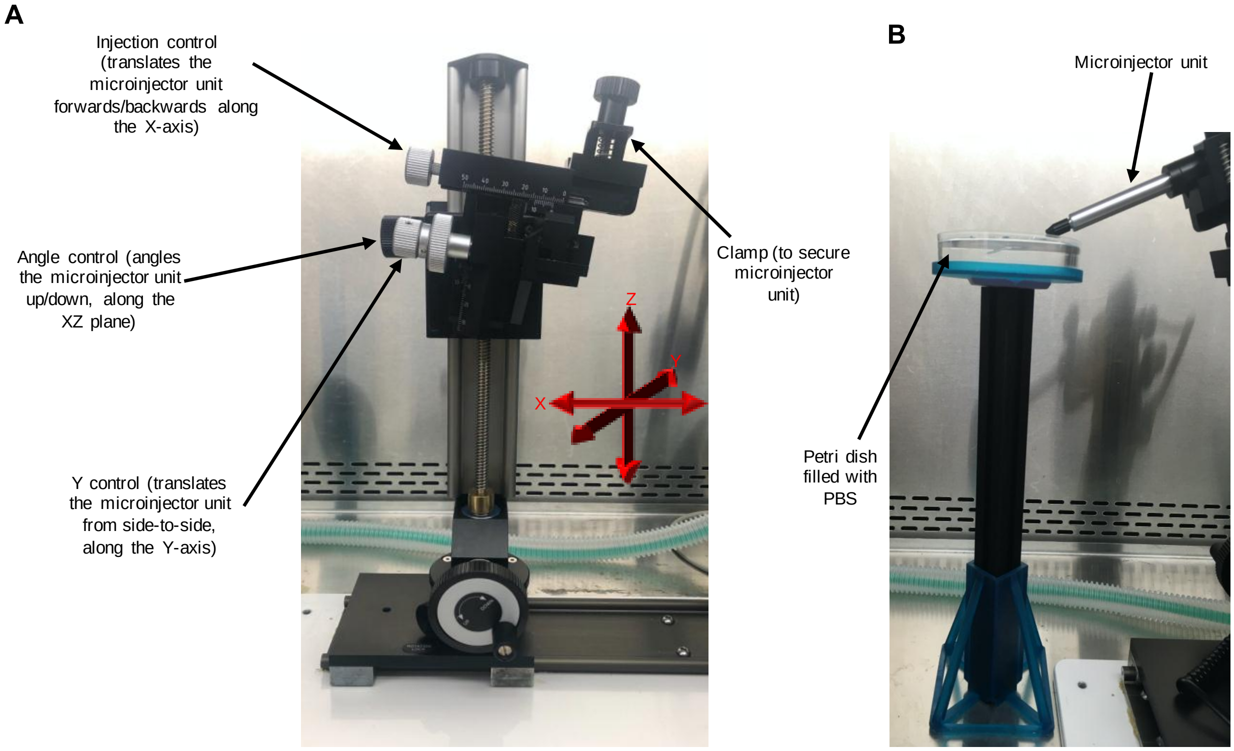

Fig. S3. Microinjection setup. A: Injector mount to secure the microinjector unit and align the needle to the imaging plane. B: 3D-printed petri dish holder. The microinjection needle was kept submerged in PBS for the duration of the surgical procedure, except when performing embolizations.

Disease Models & Mechanisms • Supplementary information

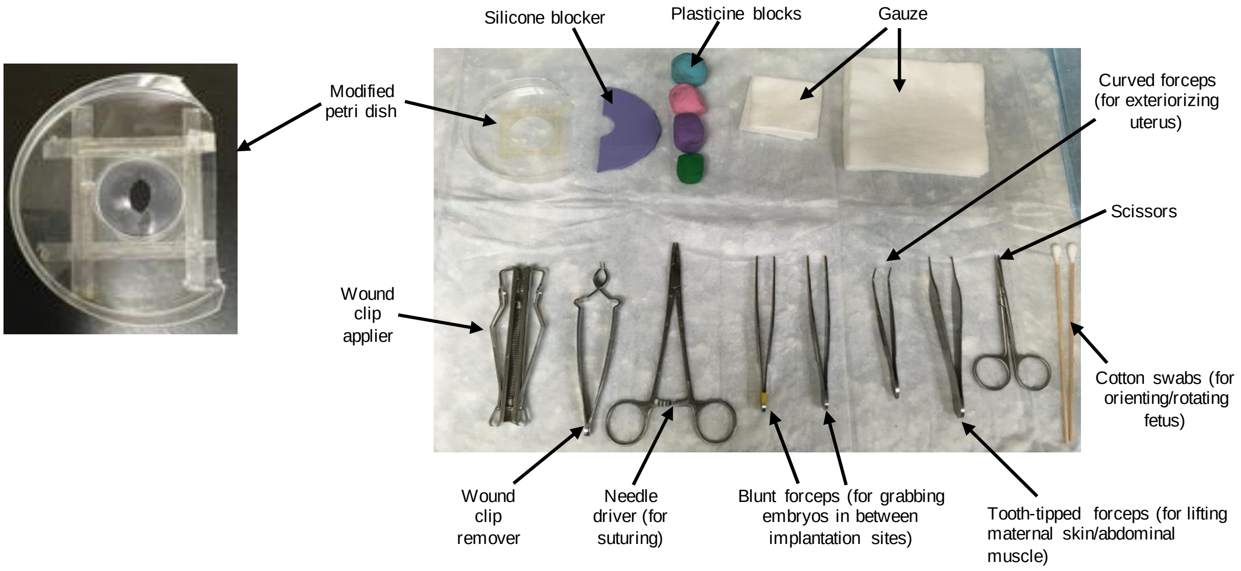

Fig. S4. Materials used during the surgical procedure. The closeup of the modified petri dish shows the oval shaped slit and the absence of the petri dish wall from one side, the removal of which was necessary for advancing the microinjection needle forward in a horizontal position without any impediment.

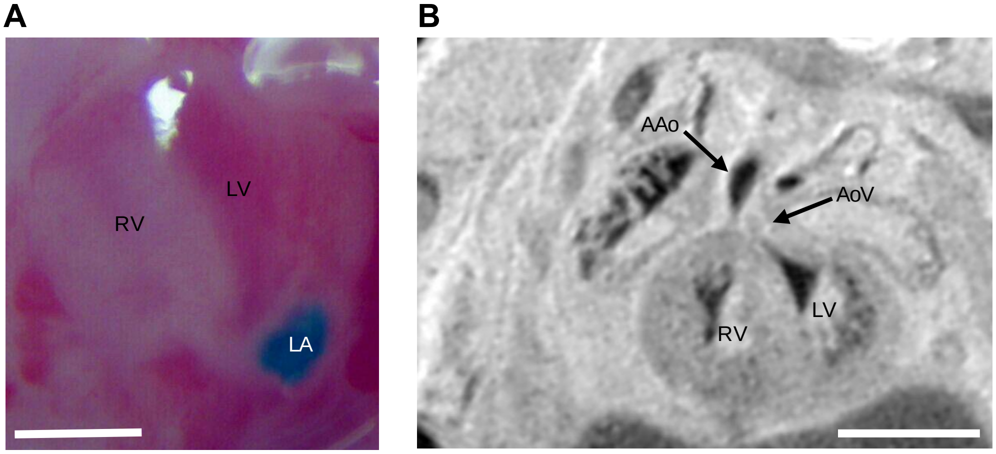

Fig. S5. Localization of the embolizing agent and morphology of the heart at E14.5. A: Posterior view of E14.5 heart showing the location of the STB embolizing agent (in blue) limited to the left atrium (injectate volume: 69 nL). B: MRI of a representative E14.5 control heart showing that the aorta is aligned with the left ventricle and that the interventricular communication is closed at this gestational age. RV: right ventricle, LV: left ventricle, LA: left atrium, AAo: ascending aorta, AoV: aortic valve. Scale bar: 1 mm.

Disease Models & Mechanisms • Supplementary information

Table S1. Fixed effects for the linear mixed effects model predicting the fetal weights for each experimental group. The degrees of freedom were estimated using the Satterthwaite approximation from the lmerTest package (Kuznetsova et al., 2017).

Term	Coefficient (g)	Standard error (g)	Degrees of freedom	t-statistic	P value
Intercept (Control Group)	1.346	4.481×10^{-2}	26.901	30.038	$< 2 \times 10^{-16}$
Surgery Littermate Group	-0.162	4.910×10^{-2}	27.257	-3.305	2.67×10^{-3}
Sham Group	-0.106	5.733×10^{-2}	49.468	-1.857	6.925×10^{-2}
Embolized Group	-0.189	5.748×10^{-2}	50.295	-3.288	1.85×10^{-3}

Table S2. Contingency table used for comparing the embolization status and aortic arch flow patterns for the Fisher's Exact test. LA: left atrium. P value = 4.993×10^{-8}

	Embolization Status	
Aortic Arch Flow Pattern	Negative (no embolization performed)	Positive (embolus inside LA)
Retrograde	0	10 fetuses
Antegrade	19 fetuses	0

Table S3. Fixed effects for the linear model predicting the ascending aorta volume for each experimental group with control and embolized groups as the reference group

Term	Coefficient (mm^3)	Standard error (mm^3)	t-statistic	P value
Intercept (Control Group)	7.677×10^{-2}	4.496×10^{-3}	17.073	$< 2 \times 10^{-16}$
Sham Group	-1.225×10^{-2}	5.981×10^{-3}	-2.048	4.91×10^{-2}
Embolized Group	-5.725×10^{-2}	6.213×10^{-3}	-9.215	2.17×10^{-10}
Intercept (Embolized Group)	1.951×10^{-2}	4.287×10^{-3}	4.552	7.73×10^{-5}
Sham	4.500×10^{-2}	5.825×10^{-3}	7.726	1.03×10^{-8}

Disease Models & Mechanisms • Supplementary information

Table S4. Fixed effects for the linear model predicting the main pulmonary artery volume for each experimental group with control and embolized groups as the reference group.

Term	Coefficient (mm^3)	Standard error (mm^3)	t-statistic	P value
Intercept (Control Group)	3.806×10^{-2}	3.390×10^{-3}	11.227	1.89×10^{-12}
Sham Group	-4.655×10^{-3}	4.590×10^{-3}	-1.014	3.18×10^{-1}
Embolized Group	1.283×10^{-2}	4.590×10^{-3}	2.796	8.81×10^{-3}
Intercept (Embolized Group)	5.089×10^{-2}	3.094×10^{-3}	16.445	$< 2 \times 10^{-16}$
Sham	-1.749×10^{-2}	4.376×10^{-3}	-3.996	3.70×10^{-4}

Supplementary References:

1. Kuznetsova, A., Brockhoff, P., and Christensen, R. (2017). lmerTest Package: Tests in Linear Mixed Effects Models. *Journal of Statistical* Software, **82**, pp.1-26.

Disease Models & Mechanisms • Supplementary information

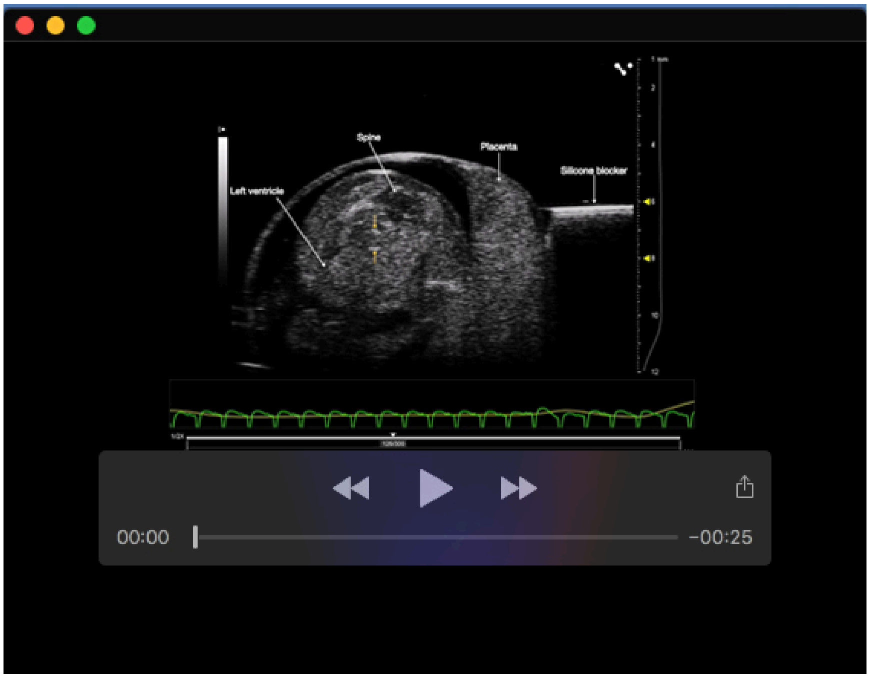

Movie 1. B-mode ultrasound showing the proper orientation of the fetal heart prior to microinjection. In this view, the highly echogenic top and bottom walls of the left atrium can be visualized as bright horizontal lines (indicated by yellow arrows).

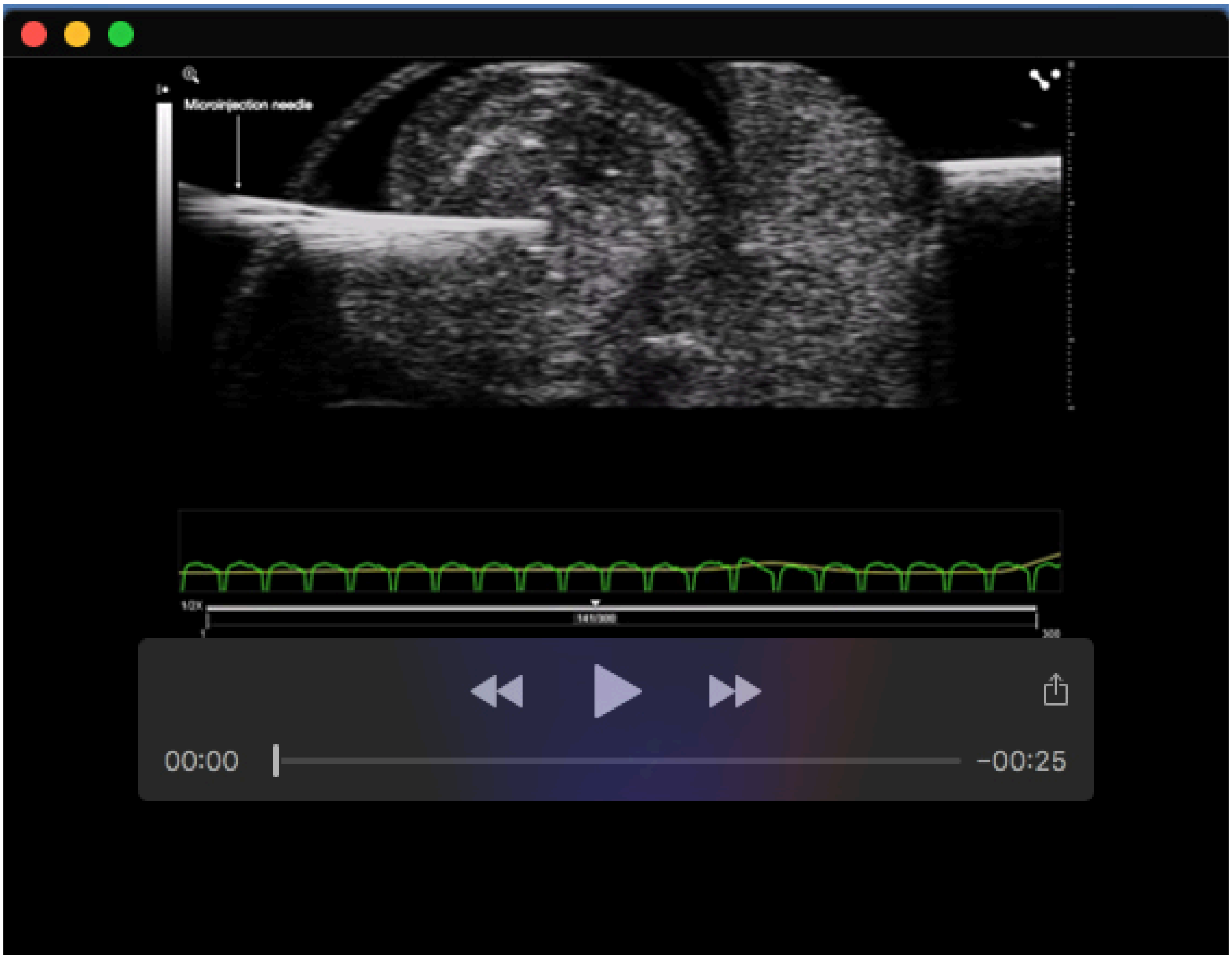

Movie 2. During the microinjection procedure, the needle is advanced through the uterus and into the fetal left atrium while avoiding critical structures such as the fetal limbs and the vitelline vessels. In this view, the needle tip is in the same imaging plane as the echogenic left atrium walls and appears bright on B-mode ultrasound. Prior to delivery of the embolizing agent, the needle tip should be localized centrally within the left atrium cavity, surrounded by both the top and bottom walls of the left atrium.

Disease Models & Mechanisms • Supplementary information

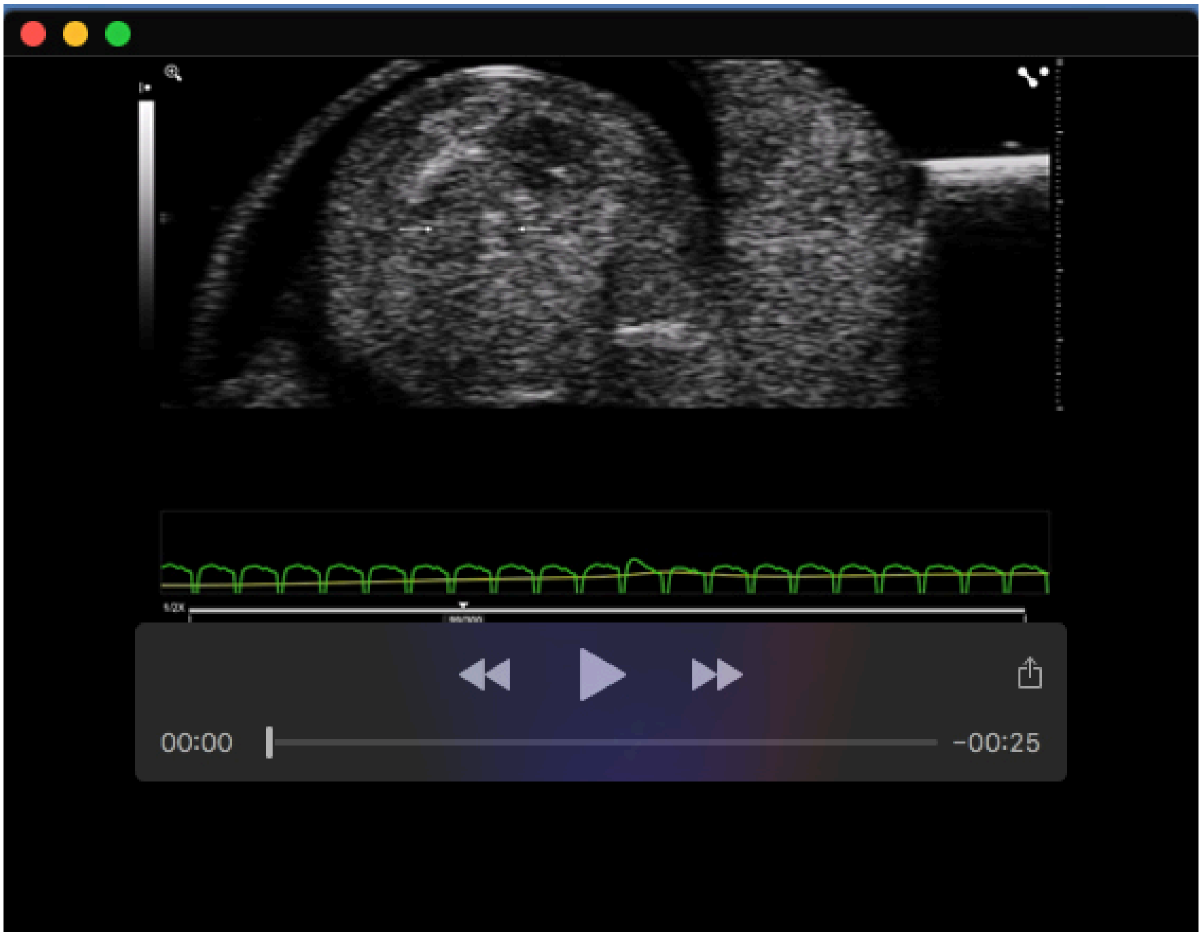

Movie 3. Post-embolization, a hypoechogenic black mass can be observed moving inside the left atrium horizontally, in between the white arrows, confirming that the embolization was positive and that the embolus was limited to the left atrium. In this imaging plane, vessels arising from the vitelline circulation (encircled) can also be observed as moving bright white speckles (moving blood). These vessels should be avoided during needle advancement (by translating the needle out of the imaging plane) in order to prevent fetal demise.
